# Radiologists’ Expectations of Artificial Intelligence in Pancreatic Cancer Imaging: How Good is Good Enough?

**DOI:** 10.1097/RCT.0000000000001503

**Published:** 2023-07-28

**Authors:** Linda C. Chu, Taha Ahmed, Alejandra Blanco, Ammar Javed, Edmund M. Weisberg, Satomi Kawamoto, Ralph H. Hruban, Kenneth W. Kinzler, Bert Vogelstein, Elliot K Fishman

**Affiliations:** 1 The Russell H. Morgan Department of Radiology and Radiological Science, Johns Hopkins Hospital, Baltimore, Maryland; 2 Department of Surgery, New York University Grossman School of Medicine, New York, NY; 3 Sol Goldman Pancreatic Cancer Research Center, Department of Pathology, Johns Hopkins University School of Medicine, Baltimore, MD; 4 Sidney Kimmel Comprehensive Cancer Center, Johns Hopkins University School of Medicine, Baltimore, MD.

**Keywords:** AI, pancreas, survey, radiologist expectations

## Abstract

**Background:**

Existing (artificial intelligence) tools in radiology are modeled without necessarily considering the expectations and experience of the end user - the radiologist. The literature is scarce on the tangible parameters that artificial intelligence capabilities need to meet for radiologists to consider them useful tools.

**Objective:**

To explore radiologists’ attitudes towards artificial intelligence tools in pancreatic cancer imaging and to quantitatively assess their expectations of these tools.

**Methods:**

A link to the survey was posted on the www.ctisus.com website, advertised in the www.ctisus.com email newsletter, and publicized on LinkedIn, Facebook, and Twitter accounts. This survey asked participants about their demographics, practice, and current attitudes toward artificial intelligence. They were also asked about their expectations of what constitutes a clinically useful artificial intelligence tool. The survey consisted of 17 questions, which included 9 multiple choice questions, 2 Likert scale questions, 4 binary (yes/no) questions, 1 rank order question, and 1 free text question.

**Results:**

A total of 161 respondents completed the survey, yielding a response rate of 46.3% of the total 348 clicks on the survey link.. The minimum acceptable sensitivity of an AI program for the detection of pancreatic cancer chosen by most respondents was either 90% or 95% at a specificity of 95%. The minimum size of pancreatic cancer that most respondents would find an artificial intelligence useful at detecting was 5 mm. Respondents preferred artificial intelligence tools that demonstrated greater sensitivity over those with greater specificity. Over half of respondents anticipated incorporating artificial intelligence tools into their clinical practice within the next 5 years.

**Conclusion:**

Radiologists are open to the idea of integrating AI-based tools and have high expectations regarding the performance of these tools. Consideration of radiologists’ input is important to contextualize expectations and optimize clinical adoption of existing and future AI tools.

## INTRODUCTION

Pancreatic cancer (pancreatic ductal adenocarcinoma, PDAC) is the third leading cause of cancer-related death in the United States (1). One of the strongest drivers of poor outcomes in these patients is the asymptomatic nature of the disease resulting in diagnosis at an advanced stage in most patients (2). Furthermore, subtle features can be overlooked on imaging in patients with low-stage disease.

Computed tomography (CT) is currently employed as the primary imaging technique to assess PDAC, with a reported sensitivity ranging from 76% to 96% (3–9). Early signs of PDAC can be elusive due to their subtle CT features and can be missed even by experienced radiologists. Retrospective reviews have identified subtle early indicators, such as irregular pancreatic parenchyma and loss of normal fatty marbling, to be present on CT up to 34 months prior to PDAC diagnosis (10). Artificial intelligence (AI) guided automated analysis of these imaging features presents a promising avenue for computer-aided PDAC diagnosis.

Recent studies have explored the application of AI to perform auto-detection of pancreatic cancer from computed tomography (CT) scans. As of the end of 2022, there were over 200 radiology-related AI algorithms that had been approved by the United States Food and Drug Administration (FDA) (11). These programs, often designed by radiologists and computer scientists, are based on mathematical modeling of an algorithm without necessarily considering the expectations or experience of the end user, i.e., the radiologist (12).

The aims of this study were to survey radiologist’s perspectives regarding the application of AI in the management of PDAC, and to determine the level of performance and essential features that these AI-based systems should have before radiologists will adopt them clinically.

## MATERIAL AND METHODS

The survey was considered exempt by the Johns Hopkins Institutional Review Board and no informed consent was required. A survey consisting of 17 questions was administered via Qualtrics^XM^ (Qualtrics, Provo, UT) for a four-week period between November and December 2020. The survey underwent several developmental iterations and was reviewed by 2 experienced radiologists for relevance and clarity. The link to the survey was posted on the www.ctisus.com website, advertised in the www.ctisus.com email newsletter, and publicized on LinkedIn, Facebook, and Twitter accounts. The email advertisement was sent on Days 1 and 15 of the study. Response collection was anonymous. Responses from all self-reported radiologists were considered eligible. The total number of radiologists who completed each question varied depending on the question asked; therefore, denominators and percentages were reported based on the number of responses for each question. All descriptive statistical analysis was performed using IBM SPSS version 27.

### Survey characteristics

The survey consisted of 17 questions, which included 9 multiple choice questions, 2 Likert scale questions, 4 binary (yes/no) questions, 1 rank order question, and 1 free text question (Supplemental Digital Content 1). The Likert and binary questions contained more than one question stem (Table S1). To assess the preferred level of performance offered by the AI-based systems, five choices each were provided for sensitivity (80%, 85%, 90%, 95%, and 99%) and specificity (75%, 80%, 85%, 90%, and 95%). Perceived usefulness of specific features of the system were assessed on a 5-point Likert Scale for the following options: PDAC only, pancreatic solid and cystic neoplasms, pancreatic neoplasms and pancreatitis, classify as abnormal but not localize, localize abnormality but not diagnosis, and locate abnormality and provide diagnosis.

## RESULTS

The survey invitation email was sent to 7,929 radiologists and technologists on the www.ctisus.com mailing list. The invitation stated that the survey was intended for radiologists only and received a total of 173 clicks on the survey link. The advertisement on LinkedIn received 25 clicks, 79 clicks on Facebook, and 71 clicks on Twitter. Of the 348 clicks on the survey page, 161 (46.3%) respondents completed the survey. Respondent self-reported characteristics are summarized in Table 1. Respondents varied in age, years in practice, and geographic location. Approximately two-thirds of the respondents were male (66.9%). Approximately one-third of respondents were in private practice (31.5%), one-third in academics (32.7%), and one-third in a combination of private practice and academics (35.9%).

**Figure 1.**
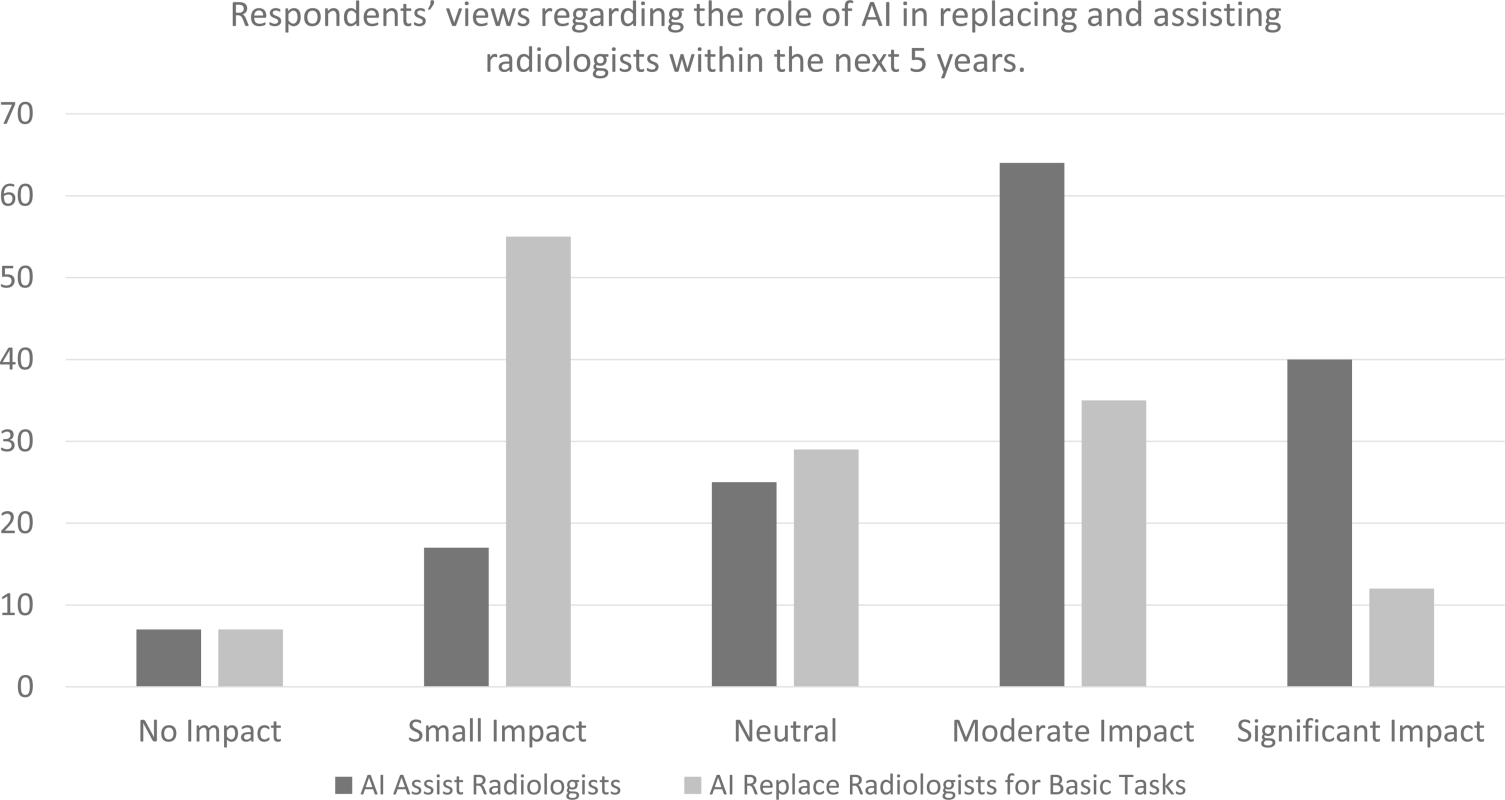
Respondents’ views regarding the role of AI in replacing and assisting radiologists within the next 5 years.

Respondents rated the potential role of AI in radiology within the next 5 years on a scale of 1 to 5 (1 = no impact, 5 = significant impact). The median score for impact of AI assisting radiologists in image interpretation within the next 5 years was 4 (moderate impact). The median score for the impact of AI replacing radiologists in basic image interpretation tasks within the next 5 years was 3 (neutral) (Figure 1). Over half of the respondents (54.6%, 83/152) believed that their group, hospital, or practice will adopt AI into their practice over the next 5 years.

The respondents’ expectations of the diagnostic performance of AI programs are summarized in Table 2. The minimum sensitivity thresholds for a useful AI program for detection of pancreatic cancer chosen by 49 respondents was 95% (49/143, 34%), while 41 respondents chose 90% (41/143, 29%). The majority (90 of 143, 63%) of respondents thereby considered the minimally acceptable sensitivity to be 90%. Similarly, most respondents (78/142, 55%) found the minimally acceptable specificity to be 95%. The majority of respondents (82/143, 57.3%) chose 5 mm as the minimum size threshold for detection of pancreatic cancer.

**Figure 2.**
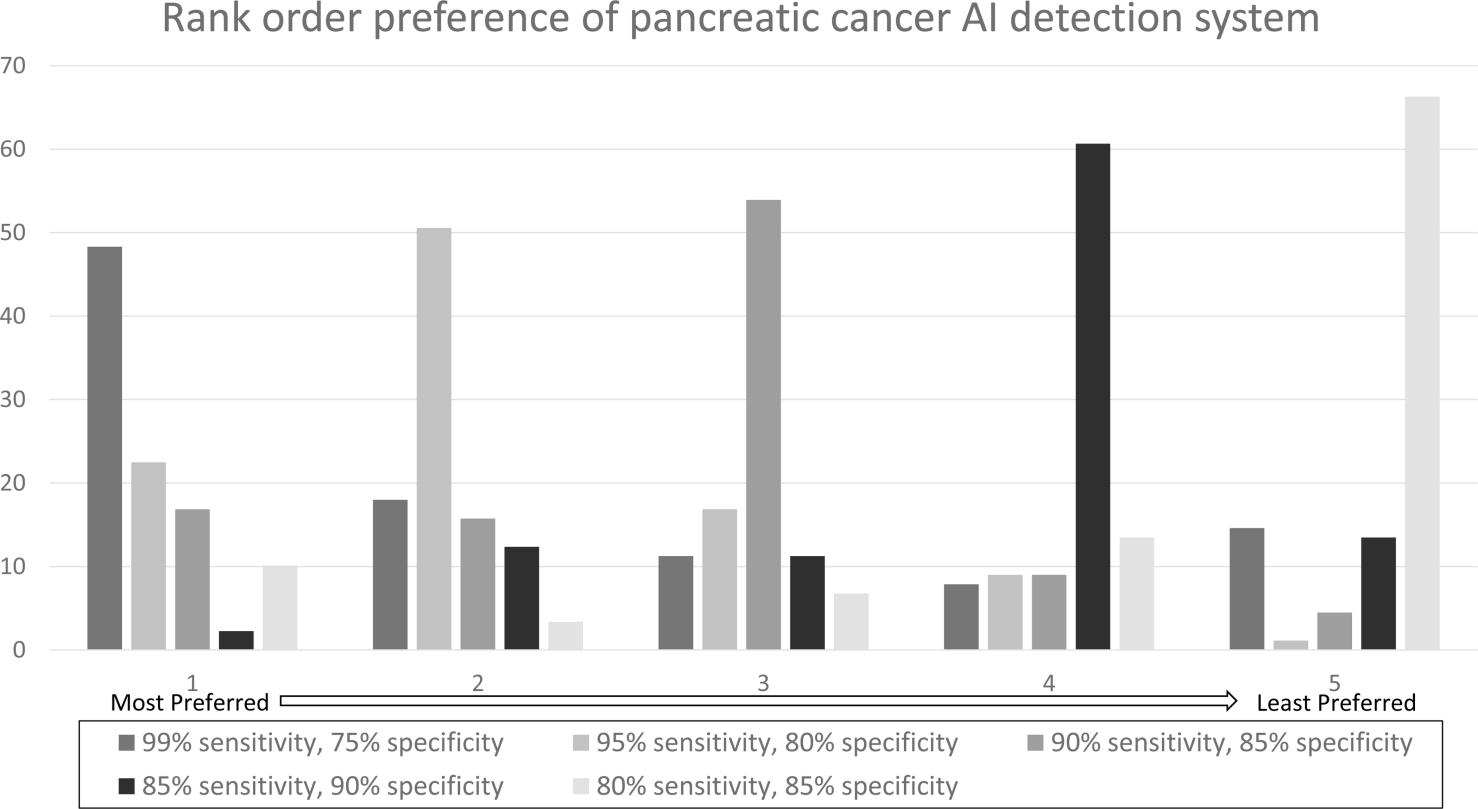
Rank order preference of pancreatic cancer AI detection system. Respondents were asked to rank their most preferred (1) to least preferred (5) system with the hypothetical sensitivities and specificities.

**Figure 3.**
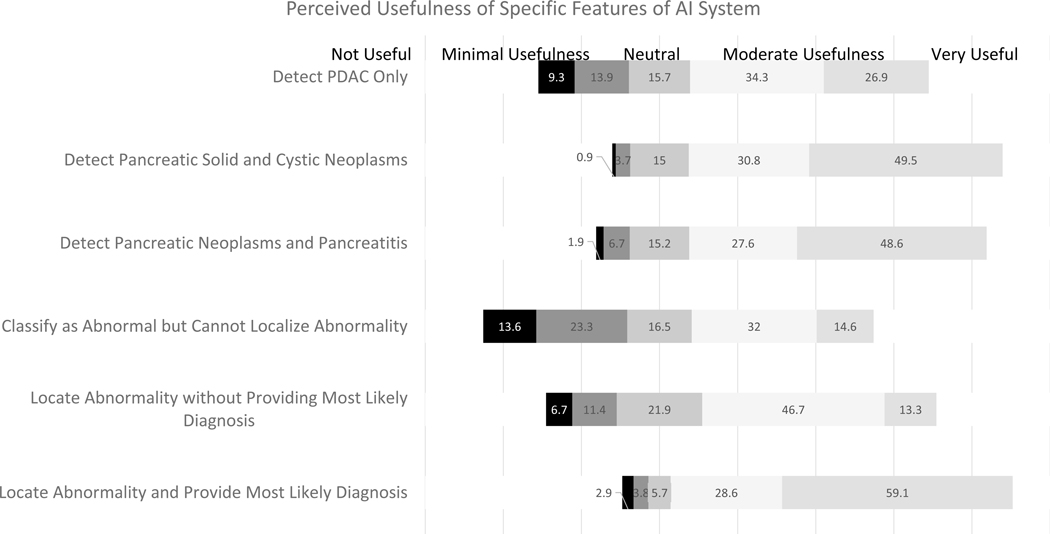
Perceived Utility of Specific Features of Artificial Intelligence System on the 5-Point Likert Scale

The respondents also rated the usefulness of a hypothetical AI system in the detection of pancreatic cancer in a binary fashion (yes/no) (Table 3). An AI system capable of detecting 100% of pancreatic cancer was deemed useful by 90% (91/101) of respondents. 84% (84/100) of respondents rated an AI system capable of detecting 95% of pancreatic cancer and missing 5% of cases as useful, while 57% (57/100) of respondents rated an AI system capable of detecting 90% of pancreatic cancer and missing 10% of cases as useful. Respondents ranked AI systems with 99% sensitivity and 75% specificity as their most preferred accuracy when asked to rank systems with varying cutoffs for sensitivities and specificities (Figure 2). When asked to rank the usefulness of specific features of AI on a 5-Point Likert Scale, most participants ranked the AI tool’s ability to diagnose and localize abnormality highest, followed by the ability to diagnose and localize pancreatic solid and cystic neoplasms (Figure 3).

## DISCUSSION

Recent studies have demonstrated the utility of integrating AI-based systems in the radiological assessment of diseases (13–15). However, despite advances in AI and predictions of widespread implementation, only 30% of current radiology practices report utilizing these tools (16). The reasons for the lack of more widespread adoption of AI are multifactorial but may be broadly categorized into issues stemming from the lack of clinical validation of AI tools, questions regarding the financial sustainability of AI tools, and legal hurdles associated with integration of AI into healthcare (17). While there has been work conducted on the general willingness of radiologists to embrace AI, research on the tangible thresholds that AI capabilities need to meet for radiologists to consider clinical adoption remains limited. Our study aimed to capture radiologists’ perceptions of what constitutes a clinically useful AI-based system in pancreatic cancer imaging.

The results of our survey demonstrated that radiologists have high thresholds for AI performance that they would find useful for adopting in their clinical practice. A majority reported that they would not utilize any tool with a sensitivity of less than 90% and a specificity <95%. Of note, our respondents rated AI tools with higher sensitivity and lower specificity more favorably than those with higher specificity and lower sensitivity. These results suggest that radiologists perceive these AI-based tools to be most useful for screening purposes that provide them with the safety net of a second read to rule out potentially missed diagnoses rather than a tool that rules in a particular diagnosis with a high level of certainty.

This survey is of particular relevance in diseases that are often missed on assessment, including early-stage PDAC (13). Notably, previous studies have attempted to gauge the ability of radiologists to detect pancreatic lesions on CT and found their reported sensitivity to range between 74%–96% (4,6,8,9,18–20). When compared with the results from our survey, this indicates that the minimum standard that radiologists expect from AI may be higher than what radiologists themselves actually achieve in practice. Similarly, the minimum size PDAC threshold that over half of the surveyed radiologists would consider an AI useful at detecting was 5 mm. Pancreatic lesions of this size are notoriously difficult to detect on CT, even when the imaging is reviewed by experienced radiologists. Prior studies have found that nearly 40% of pancreatic cancers under 2 cm are missed by radiologists on CT (21). As such, it is probable that a pancreatic AI tool not entirely meeting the minimum expectations of our respondents could still improve reporting among radiologists. However, based on the results of this survey, it is likely that until these AI systems can detect such smaller lesions with high accuracy, their adoption in the clinical setting will remain minimal.

Over the past few years, several studies have gauged the attitude of radiologists regarding fear of AI replacing their roles. Interestingly, in our survey, only 8% of respondents believed that AI would have a significant impact on replacing radiologists, even for basic imaging tasks, within the next 5 years. This contrasts with earlier surveys in which up to 38%–61% of respondents expressed concern regarding the replacement of radiologists by AI (22,23). Our results are likely reflective of the broader paradigm shift that has taken place regarding the role of AI within the radiology community in recent years. As initial fears of replacement by AI have been tempered, apprehension regarding replacement has evolved into optimism about a mutually beneficial co-dependence. Indeed, AI seems poised to revolutionize the practice of radiology, but not replace radiologists. Instead, as stated by Dr. Curtis Langlotz “Radiologists who use AI will replace radiologists who don’t,” (24).

Recent studies on AI in pancreatic imaging have reported the ability of AI tools to independently diagnose pancreatic lesions with a sensitivity and specificity of up to 90% and 93%, respectively (13). Therefore, it seems likely that within the next 5 years, the expectations of even the most rigorous respondents will be on track to be met. Nevertheless, optimism surrounding the results of independent AI studies must be cautiously tempered, as it is yet to be seen how the results of AI algorithms from these studies generalize to commercially available platforms and daily practice in the broad population. In addition, claims about performance metrics should be duly scrutinized, as metrics like specificity and sensitivity can significantly vary depending on the study population and institutional characteristics, including protocols for study acquisition and testing methods. It is also doubtful that commercial AI software will be optimized at launch. Rather, it is most likely that the effective implementation of AI will consist of a continually self-correcting process involving real-time feedback between AI developers and radiologists, much like the process through which electronic medical record (EMR) software was gradually optimized. In the case of EMR, failure to consult physicians during development led to years of problems following implementation (25,26). Consulting radiologists in the development of AI tools would therefore be prudent and may preemptively address similar issues that arise during clinical adoption of AI tools.

Even if the technical needs of our respondents from AI were to eventually be fully met, an important consideration when evaluating the integration of AI tools into clinical practice is that of the costs and increased workload associated with the acquisition and maintenance of AI tools. Improved diagnostic accuracy without direct cost or time savings attributable to the software may not be sufficient to drive software adoption in mature healthcare economies. These concerns were seen in a previous survey of radiologists’ attitudes towards AI, wherein 39% of respondents reported cost and increased workload as serious potential drawbacks of workplace AI integration (27). Amidst our current landscape of high volumes and monitored turnaround times, any decrease in efficiency will likely be hard to accept on an individual level. With each false positive finding flagged by AI adding to radiologists’ reading time and mouse mileage, it is understandable why the radiologists we surveyed would tolerate only a low number of false positives. This issue is further exacerbated in practices adopting fee-for-service models, where quality improvement in the absence of cost savings or increased revenue can deter implementation (28). Consequently, AI companies often focus on improving efficiency through measures such as increasing acquisition or interpretation speed (29,30). These companies operate on the rationale that saving time translates directly into cost savings (12). One possible solution to this could be to develop a separate reimbursement model or adopt the billing framework that already exists for computer aided detection in breast imaging. Studies focusing on what stakeholders consider the optimal balance between cost, time, and added diagnostic value of AI will be essential for guiding future development of a potential reimbursement model.

Our study has several limitations. First, the survey’s low response rate limits the generalizability of the findings across all radiologists. Furthermore, the majority of the responders were in the early years of their practice. The adoption of these technologically complex systems by senior radiologists warrants further investigation. There may be a selection bias in that younger radiologists who view AI more favorably may be more likely to answer a survey on AI performance. Lastly, the administrators in radiology departments and practices were not surveyed. They are key stakeholders in making decisions regarding the uptake of new technology. Their attitudes and concerns toward the adoption of these AI systems in clinical practice need to be explored further.

In conclusion, this study demonstrates that radiologists are open to the idea of integrating AI-based tools as long as they meet high though probably attainable performance criteria. We believe that, based on continuing progress in the technical capabilities of AI as well as instrumentation, and the results of this survey, the clinical implementation of AI technology for the detection of pancreatic cancer is a worthy and feasible goal. Future studies should transition towards investigating the preliminary experiences of current radiology AI users to guide further development of AI and to encourage AI adoption among practices not currently using AI tools.

## Supplementary Material

Supplemental Digital Content 1 **Supplemental Digital Content 1.** Survey questionnaire.

## Figures and Tables

**Table 1.** Characteristics of the Respondents

Respondent Characteristics	Number (%)
Age (years) <30 31–40 41–50 51–60 >60	15/161 (9.3) 35/161 (21.7) 42/161 (26.1) 42/161 (26.1) 27/161 (16.8)
Sex Male Female Prefer not to answer	107/160 (66.9) 51/160 (31.9) 2/160 (1.3)
Years in practice (after completion of residency and/or fellowship) 1–10 11–20 21–30 31–40 >40	62/160 (38.8) 35/160 (21.9) 40/160 (25.0) 21/160 (13.1) 2/160 (1.3)
Location of practice United States North American outside of United States South America Europe Asia Oceania Africa	39/159 (24.5) 12/159 (7.6) 20/159 (12.6) 61/159 (38.4) 26/159 (16.4) 0/159 (0) 1/159 (0.6)
Practice environment Private practice Academic Combination of private practice and academic	50/159 (31.5) 52/159 (32.7) 57/159 (35.9)
Percent of time spent in clinical abdominal imaging 0–20 21–40 41–60 61–80 81–100	17/160 (10.6) 31/160 (19.4) 53/160 (33.1) 41/160 (25.6) 18/160 (11.3)

**Table 2.** Respondents’ Expectations for the Pancreatic Cancer Artificial Intelligence Program

Topic	Number (%)
Minimum sensitivity (%) of A.I. program that they will consider using: >99 >95 >90 >85 >80	18/143 (12.6) 49/143 (34.3) 41/143 (28.7) 20/143 (14.0) 15/143 (10.5)
Number of false positives they are willing to tolerate per pancreatic cancer detected: <5 5–10 10–15 15–20 >20	78/142 (54.9) 39/142 (27.5) 15/142 (10.6) 6/142 (4.2) 4/142 (2.8)
Minimum size threshold (mm) that the program should be able to detect: 3 5 10 15 >20	32/143 (22.4) 82/143 (57.3) 25/143 (17.5) 4/143 (2.8) 0/143 (0)

**Table 3.** Perceived Usefulness of Artificial Intelligence Program given Hypothetical System Performance

Hypothetical System Performance	Rated as Useful (%)
Percentage of pancreatic cancer detected by AI 100% 95% 90% 85% 80%	91/101 (90.1) 84/100 (84.0) 57/100 (57.0) 28/97 (28.9) 18/96 (18.8)
Number of false positives provided by AI for second look 0 false positives/100 normal pancreata 5 false positives/100 normal pancreata 10 false positives/100 normal pancreata 15 false positives/100 normal pancreata 20 false positives/100 normal pancreata	66/90 (73.3) 86/100 (86.0) 54/96 (56.3) 17/91 (18.7) 15/90 (16.7)
