# Supplementary material for: Radiologists’ Expectations of Artificial Intelligence in Pancreatic Cancer Imaging: How Good is Good Enough?
Source: J Comput Assist Tomogr. Author manuscript; Available in PMC 2024 Jul 28. (PMC10823576; doi:10.1097/RCT.0000000000001503)
Supplement: Supplemental Digital Content 1 — Survey questionnaire. [file NIHMS1895288-supplement-Supplemental_Digital_Content_1.docx]

Pancreas AI Survey

Thank you for participating in our survey on the application of artificial intelligence (AI) in pancreatic cancer imaging. We would like to determine the level of performance and essential features that an AI system should have before radiologists will consider using it clinically. The results generated from this survey will serve as a key source of input as we develop any future pancreas AI prototypes.

The survey takes less than 10 minutes to complete. Your participation is entirely voluntary, and all responses are confidential and de-identified. By taking this survey, you consent to having your responses included in this study.

1. What is your age range (years)?
   1. <30
   2. 31–40
   3. 41–50
   4. 51–60
   5. >60
2. What is your birth-assigned sex?
   1. Male
   2. Female
   3. Prefer not to answer
3. How many years have you been in practice (after completion of residency and/or fellowship)?
   1. 1–10
   2. 11–20
   3. 21–30
   4. 31–40
   5. >40
4. Which part of the world do you practice in?
   1. United States
   2. North America outside of United States
   3. South America
   4. Europe
   5. Asia
   6. Oceania
   7. Africa
5. How would you describe your practice environment?
   1. Private practice
   2. Academic
   3. A combination of private practice and academic
6. How much of your time is spent in clinical abdominal imaging?
   1. 0–20%
   2. 21–40%
   3. 41–60%
   4. 61–80%
   5. 81–100%
7. What is your view about the potential role of artificial intelligence in radiology within the next 5 years (scale 1–5, 1 = No impact, 2 = Small impact, 3 = Neutral, 4 = Moderate impact, 5 = Significant impact)?

AI assisting radiologists in image interpretation:

1 2 3 4 5

AI replacing radiologists in basic image interpretation tasks:

1 2 3 4 5

1. Do you believe your group, hospital, or practice will adopt AI into your practice in the next 5 years?

Yes No

1. In detection of pancreatic cancer, what is the minimum sensitivity of an AI program that you will consider using?
2. >99%
3. >95%
4. >90%
5. >85%
6. >80%
7. How many false positives are you willing to tolerate per pancreatic cancer detected?
8. <5
9. 5–10
10. 10–15
11. 15–20
12. >20
13. What is the minimum size threshold that the program should be able to detect?
14. 3 mm
15. 5 mm
16. 10 mm
17. 15 mm
18. 20 mm
19. The following questions ask about the sensitivity of AI system performance and whether you will use the AI system to aid your diagnosis.

AI can detect 100% of pancreatic cancer:

Useful Not useful

AI can detect 95% of pancreatic cancer but misses 5% of cases:

Useful Not useful

AI can detect 90% of pancreatic cancer but misses 10% of cases:

Useful Not useful

AI can detect 85% of pancreatic cancer but misses 15% of cases:

Useful Not useful

AI can detect 80% of pancreatic cancer but misses 20% of cases:

Useful Not useful

1. If for every 100 normal pancreata analyzed, the AI suggests the provided number of false positives for second look, do you find the AI system useful?

0 cases:

Useful Not useful

5 cases:

Useful Not useful

10 cases:

Useful Not useful

15 cases:

Useful Not useful

20 cases:

Useful Not useful

1. Please rank order performance of pancreatic cancer detection AI that you will find most useful (1 = Most preferred, 5 = Least preferred)
   1. 99% sensitivity, 75% specificity
   2. 95% sensitivity, 80% specificity
   3. 90% sensitivity, 85% specificity
   4. 85% sensitivity, 90% specificity
   5. 80% sensitivity, 95% specificity
2. The following questions ask about perceived usefulness of specific features of the AI system (1 = Not useful, 2 = Minimal usefulness, 3 = Neutral, 4 = Moderate usefulness, 5 = Very useful).

AI system can detect pancreatic ductal adenocarcinoma only (no other pancreatic pathology):

1 2 3 4 5

AI system can detect pancreatic solid and cystic neoplasms:

1 2 3 4 5

AI system can detect pancreatic neoplasms and pancreatitis:

1 2 3 4 5

AI system can classify the case as abnormal but cannot localize the abnormality:

1 2 3 4 5

AI system can locate the abnormality but cannot provide the most likely diagnosis:

1 2 3 4 5

AI system can locate the abnormality and provide the most likely diagnosis:

1 2 3 4 5

1. Do you consider the following features as major potential advantages of AI-based aids to radiologists’ diagnoses?

Occasionally detecting a cancer that was missed by a radiologist, allowing earlier diagnosis?

Important Not important

Adding independent evidence that a suspicious area is indeed worthy of further evaluation?

Important Not important

Adding independent evidence that a suspicious area is not likely to represent pancreatic cancer?

Important Not important

Providing a “second unbiased look” that can be included in the report, and potentially protecting radiologists from legal action?

Important Not important

1. Any additional comments? (free text)
